# *catena*-Poly[[triphenyl­tin(IV)]-μ-5-amino-2-nitro­benzoato-κ^2^ *O* ^1^:*O* ^1′^]

**DOI:** 10.1107/S1600536811033332

**Published:** 2011-08-27

**Authors:** Yip-Foo Win, Chen-Shang Choong, Siang-Guan Teoh, Ching Kheng Quah, Hoong-Kun Fun

**Affiliations:** a Department of Chemical Science, Faculty of Science, Universiti Tunku Abdul Rahman, Perak Campus, Jalan Universiti, Bandar Barat, 31900 Kampar, Perak, Malaysia; b School of Chemical Sciences, Universiti Sains Malaysia, 11800 USM, Penang, Malaysia; c X-ray Crystallography Unit, School of Physics, Universiti Sains Malaysia, 11800 USM, Penang, Malaysia

## Abstract

The title compound, [Sn(C_6_H_5_)_3_(C_7_H_5_N_2_O_4_)]_*n*_, forms polymeric chains along [010]. The Sn^IV^ ion is five-coordinated in a distorted trigonal–bipyramidal geometry by two monodentate carboxyl­ate groups and three phenyl rings. The axial sites are occupied by the O atoms of two symmetry-related carboxyl­ate groups [O—Sn—O = 170.88 (3)°]. The benzene ring of the 5-amino-2-nitro­benzoate ligand forms dihedral angles of 82.92 (6), 81.10 (6) and 83.54 (6)° with respect to the three phenyl rings. In the crystal, the chains are linked by inter­molecular N—H⋯O and weak C—H⋯O inter­actions into a three-dimensional network. The crystal structure is further stabilized by weak inter­molecular C—H⋯π inter­actions.

## Related literature

For general background to and the coordination environment of triphenyl­tin(IV) carboxyl­ate complexes, see: Yeap & Teoh (2003 ▶); Win *et al.* (2006 ▶, 2008 ▶, 2011*a* ▶,*b* ▶,*c* ▶). For standard bond-length data, see: Allen *et al.* (1987 ▶). For the stability of the temperature controller used in the data collection, see: Cosier & Glazer (1986 ▶).
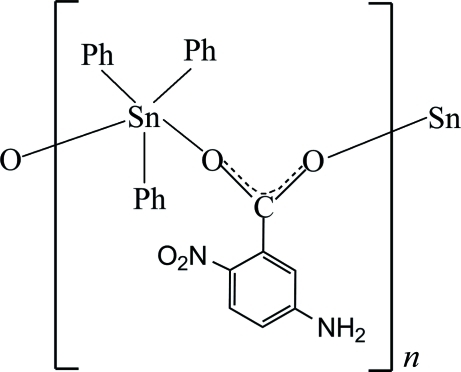


## Experimental

### 

#### Crystal data

[Sn(C_6_H_5_)_3_(C_7_H_5_N_2_O_4_)]
*M* *_r_* = 531.12
Monoclinic, 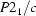

*a* = 10.9752 (1) Å
*b* = 11.8342 (1) Å
*c* = 17.4160 (2) Å
β = 102.164 (1)°
*V* = 2211.25 (4) Å^3^
*Z* = 4
Mo *K*α radiation
μ = 1.19 mm^−1^
*T* = 100 K
0.37 × 0.25 × 0.22 mm

#### Data collection

Bruker SMART APEXII CCD area-detector diffractometer
Absorption correction: multi-scan (*SADABS*; Bruker, 2009 ▶) *T*_min_ = 0.671, *T*_max_ = 0.783
27219 measured reflections
7981 independent reflections
7370 reflections with *I* > 2σ(*I*)
*R*_int_ = 0.017

#### Refinement

*R*[*F* ^2^ > 2σ(*F* ^2^)] = 0.018
*wR*(*F* ^2^) = 0.046
*S* = 1.07
7981 reflections
297 parameters
H atoms treated by a mixture of independent and constrained refinement
Δρ_max_ = 0.51 e Å^−3^
Δρ_min_ = −0.54 e Å^−3^

### 

Data collection: *APEX2* (Bruker, 2009 ▶); cell refinement: *SAINT* (Bruker, 2009 ▶); data reduction: *SAINT*; program(s) used to solve structure: *SHELXTL* (Sheldrick, 2008 ▶); program(s) used to refine structure: *SHELXTL*; molecular graphics: *SHELXTL*; software used to prepare material for publication: *SHELXTL* and *PLATON* (Spek, 2009 ▶).

## Supplementary Material

Crystal structure: contains datablock(s) global, I. DOI: 10.1107/S1600536811033332/lh5316sup1.cif

Structure factors: contains datablock(s) I. DOI: 10.1107/S1600536811033332/lh5316Isup2.hkl

Additional supplementary materials:  crystallographic information; 3D view; checkCIF report

## Figures and Tables

**Table 1:** Hydrogen-bond geometry (Å, °) *Cg*1 and *Cg*2 are the centroids of the C1–C6 and C7–C12 phenyl rings, respectively.

*D*—H⋯*A*	*D*—H	H⋯*A*	*D*⋯*A*	*D*—H⋯*A*
N1—H2*N*1⋯O1^i^	0.85 (2)	2.498 (19)	3.0619 (14)	124.3 (15)
C5—H5*A*⋯O4^ii^	0.95	2.40	3.3288 (16)	167
C3—H3*A*⋯*Cg*2^iii^	0.95	2.58	3.4430 (14)	152
C21—H21*A*⋯*Cg*1^iv^	0.95	2.70	3.4669 (12)	138

Symmetry codes: (i) 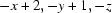; (ii) 
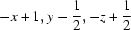
; (iii) 
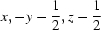
; (iv) 
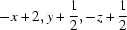
.

**Fig. 1.**
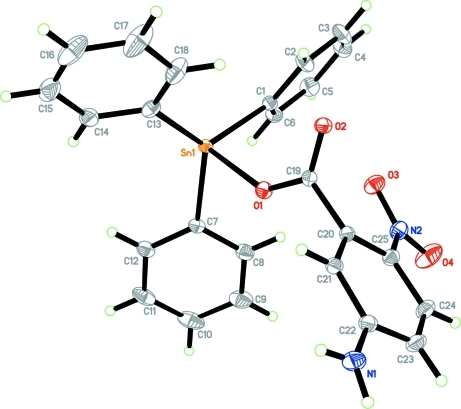
The asymmetric unit of the title compound showing 50% probability displacement ellipsoids for non-H atoms.

**Fig. 2.**
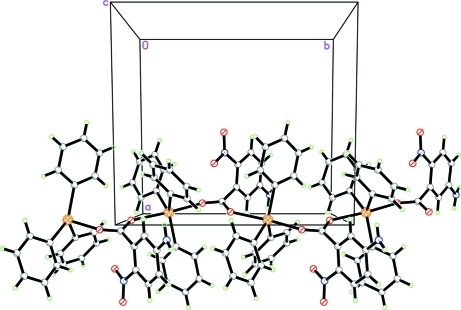
The polymeric structure of the title compound, viewed along the c axis, showing one-dimensional chains along [010].

**Fig. 3.**
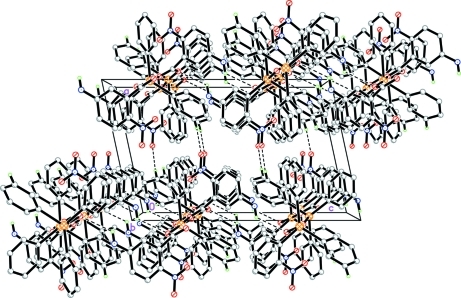
The crystal packing of the title compound, viewed along the b axis. H atoms not involved in hydrogen bonds (dashed lines) have been omitted for clarity.

## supplementary crystallographic information

### Comment

Generally, the tin(IV) atom moiety of triphenyltin(IV) carboxylate complexes could existed as four- or five-coordinated depending on the coordination manner of the carboxylate anions and the coordinating solvent (Yeap and Teoh, 2003; Win *et al.*, 2006; 2008; 2011*a*,*b*,*c*). In this study, the title complex is found to be similar to the reported structure of (2-amino-5-nitrobenzoato)triphenyltin(IV) (Win *et al.*, 2006) except that the amino group is substituted at meta-position and the nitro group is substituted at ortho-position to the benzoate group.

The asymmetric unit of the title compound is shown in Fig. 1. The overall structure consists of polymeric one-dimensional chains along [010] (Fig. 2). The Sn1 atom is five-coordinate, with a distorted trigonal-bipyramidal coordination geometry, formed by two monodentate symmetry related carboxylate groups and three phenyl rings. The axial sites are occupied by the O atoms of the two carboxylate groups [O1-Sn1-O2^i^ = 170.88 (3)°, symmetry code: (i) 2-x,-1/2+y,1/2-z], with the three phenyl rings occupying the equatorial plane. Bond lengths (Allen *et al.*, 1987) and angles are within normal ranges. The benzene ring (C20-C25) of the 5-amino-2-nitrobenzoato ligand makes dihedral angles of 82.92 (6), 81.10 (6) and 83.54 (6)° with respect to the three phenyl rings (C1-C6, C7-C12 and C13-C18).

In the crystal (Fig. 3), the polymeric one-dimensional chains are linked by intermolecular N1–H2N1···O1^iii^ and weak C5–H5A···O4^iv^ interactions into a three-dimensional network. The crystal structure is further consolidated by C21–H21A···Cg1^ii^ and C3–H3A···Cg2^v^ (Table 1) interactions, where Cg1 and Cg2 are the centroids of C1-C6 and C7-C12 phenyl rings, repectively.

### Experimental

The title complex was obtained by heating under reflux a 1:1 molar mixture of triphenyltin(IV) hydroxide (0.73 g, 2 mmol) and 5-amino-2-nitrobenzoic acid (0.36 g, 2 mmol) in methanol (50 ml) for 3 h. A clear yellow transparent solution was separated by filtration and kept in a bottle. After a few days, yellow crystals (0.48 g, 89.0 % yield) were collected. Melting point: 442-443 K. Analysis for C_25_H_20_N_2_O_4_Sn: C, 55.89; H, 3.84; N, 5.14 %. Calculated for C_25_H_20_N_2_O_4_Sn: C, 56.53; H, 3.80; N, 5.27 %.

### Refinement

Atoms H1N1 and H2N1 were located from the difference Fourier map and refined freely [N1–H = 0.852 (18) and 0.853 (19) Å]. The remaining H atoms were positioned geometrically and refined using a riding model with C–H = 0.95 Å and *U*_iso_(H) = 1.2 *U*_eq_(C). The highest residual electron density peak is located at 0.67 Å from atom C25 and the deepest hole is located at 0.71 Å from atom Sn1.

### Crystal data

**Table:**

[Sn(C_6_H_5_)_3_(C_7_H_5_N_2_O_4_)]	*F*(000) = 1064
*M**_r_* = 531.12	*D*_x_ = 1.595 Mg m^−^^3^
Monoclinic, *P*2_1_/*c*	Mo *K*α radiation, λ = 0.71073 Å
Hall symbol: -P 2ybc	Cell parameters from 9281 reflections
*a* = 10.9752 (1) Å	θ = 2.4–32.7°
*b* = 11.8342 (1) Å	µ = 1.19 mm^−^^1^
*c* = 17.4160 (2) Å	*T* = 100 K
β = 102.164 (1)°	Block, yellow
*V* = 2211.25 (4) Å^3^	0.37 × 0.25 × 0.22 mm
*Z* = 4	

### Data collection

**Table:**

Bruker SMART APEXII CCD area-detector diffractometer	7981 independent reflections
Radiation source: fine-focus sealed tube	7370 reflections with *I* > 2σ(*I*)
graphite	*R*_int_ = 0.017
φ and ω scans	θ_max_ = 32.7°, θ_min_ = 1.9°
Absorption correction: multi-scan (*SADABS*; Bruker, 2009)	*h* = −16→16
*T*_min_ = 0.671, *T*_max_ = 0.783	*k* = −17→15
27219 measured reflections	*l* = −24→26

### Refinement

**Table:**

Refinement on *F*^2^	Primary atom site location: structure-invariant direct methods
Least-squares matrix: full	Secondary atom site location: difference Fourier map
*R*[*F*^2^ > 2σ(*F*^2^)] = 0.018	Hydrogen site location: inferred from neighbouring sites
*wR*(*F*^2^) = 0.046	H atoms treated by a mixture of independent and constrained refinement
*S* = 1.07	*w* = 1/[σ^2^(*F*_o_^2^) + (0.0187*P*)^2^ + 0.9636*P*] where *P* = (*F*_o_^2^ + 2*F*_c_^2^)/3
7981 reflections	(Δ/σ)_max_ = 0.003
297 parameters	Δρ_max_ = 0.51 e Å^−^^3^
0 restraints	Δρ_min_ = −0.54 e Å^−^^3^

### Special details

**Table:**

Experimental. The crystal was placed in the cold stream of an Oxford Cryosystems Cobra open-flow nitrogen cryostat (Cosier & Glazer, 1986) operating at 100.0 (1) K.
Geometry. All esds (except the esd in the dihedral angle between two l.s. planes) are estimated using the full covariance matrix. The cell esds are taken into account individually in the estimation of esds in distances, angles and torsion angles; correlations between esds in cell parameters are only used when they are defined by crystal symmetry. An approximate (isotropic) treatment of cell esds is used for estimating esds involving l.s. planes.
Refinement. Refinement of F^2^ against ALL reflections. The weighted R-factor wR and goodness of fit S are based on F^2^, conventional R-factors R are based on F, with F set to zero for negative F^2^. The threshold expression of F^2^ > 2sigma(F^2^) is used only for calculating R-factors(gt) etc. and is not relevant to the choice of reflections for refinement. R-factors based on F^2^ are statistically about twice as large as those based on F, and R- factors based on ALL data will be even larger.

### Fractional atomic coordinates and isotropic or equivalent isotropic displacement parameters (Å^2^)

**Table:**

	*x*	*y*	*z*	*U*_iso_*/*U*_eq_	
Sn1	0.980205 (6)	0.157183 (6)	0.217102 (4)	0.01124 (2)	
O1	0.93081 (8)	0.32432 (7)	0.16122 (5)	0.01487 (14)	
O2	0.97650 (7)	0.46996 (7)	0.24440 (4)	0.01352 (14)	
O3	0.70740 (8)	0.38800 (8)	0.21137 (5)	0.02102 (17)	
O4	0.53208 (8)	0.43580 (10)	0.13585 (6)	0.0329 (2)	
N1	0.89051 (11)	0.67399 (10)	−0.04786 (6)	0.0218 (2)	
N2	0.64692 (9)	0.43960 (9)	0.15452 (6)	0.01829 (18)	
C1	0.87574 (10)	0.17504 (9)	0.30535 (6)	0.01377 (18)	
C2	0.89877 (11)	0.25962 (10)	0.36218 (7)	0.0193 (2)	
H2A	0.9597	0.3158	0.3599	0.023*	
C3	0.83296 (13)	0.26235 (12)	0.42251 (7)	0.0243 (2)	
H3A	0.8496	0.3199	0.4613	0.029*	
C4	0.74309 (12)	0.18074 (12)	0.42582 (7)	0.0247 (2)	
H4A	0.6988	0.1823	0.4672	0.030*	
C5	0.71792 (11)	0.09704 (12)	0.36888 (7)	0.0224 (2)	
H5A	0.6557	0.0419	0.3708	0.027*	
C6	0.78424 (10)	0.09427 (10)	0.30890 (7)	0.0176 (2)	
H6A	0.7670	0.0368	0.2700	0.021*	
C7	0.86334 (10)	0.08532 (9)	0.11625 (6)	0.01378 (18)	
C8	0.73920 (10)	0.12127 (11)	0.09743 (7)	0.0191 (2)	
H8A	0.7118	0.1796	0.1272	0.023*	
C9	0.65528 (12)	0.07180 (14)	0.03508 (7)	0.0276 (3)	
H9A	0.5712	0.0969	0.0222	0.033*	
C10	0.69493 (13)	−0.01430 (14)	−0.00817 (8)	0.0293 (3)	
H10A	0.6375	−0.0486	−0.0502	0.035*	
C11	0.81737 (14)	−0.05009 (11)	0.00968 (7)	0.0257 (3)	
H11A	0.8442	−0.1089	−0.0200	0.031*	
C12	0.90166 (12)	0.00017 (10)	0.07139 (7)	0.0186 (2)	
H12A	0.9861	−0.0239	0.0830	0.022*	
C13	1.17373 (10)	0.19205 (10)	0.23296 (6)	0.01591 (19)	
C14	1.25426 (11)	0.11915 (12)	0.20473 (7)	0.0212 (2)	
H14A	1.2224	0.0527	0.1770	0.025*	
C15	1.38145 (12)	0.14349 (14)	0.21712 (8)	0.0290 (3)	
H15A	1.4356	0.0938	0.1974	0.035*	
C16	1.42905 (12)	0.23968 (14)	0.25801 (10)	0.0352 (4)	
H16A	1.5155	0.2561	0.2661	0.042*	
C17	1.34983 (13)	0.31200 (13)	0.28711 (12)	0.0383 (4)	
H17A	1.3825	0.3774	0.3158	0.046*	
C18	1.22256 (11)	0.28880 (11)	0.27429 (9)	0.0271 (3)	
H18A	1.1687	0.3391	0.2938	0.032*	
C19	0.92115 (9)	0.42633 (9)	0.18082 (6)	0.01185 (17)	
C20	0.84255 (9)	0.49904 (9)	0.11779 (6)	0.01259 (17)	
C21	0.90148 (10)	0.55602 (9)	0.06630 (6)	0.01415 (18)	
H21A	0.9895	0.5512	0.0730	0.017*	
C22	0.83273 (11)	0.62117 (10)	0.00411 (6)	0.01612 (19)	
C23	0.70317 (11)	0.63147 (11)	−0.00281 (7)	0.0196 (2)	
H23A	0.6562	0.6784	−0.0425	0.024*	
C24	0.64407 (10)	0.57407 (11)	0.04748 (7)	0.0194 (2)	
H24A	0.5564	0.5806	0.0420	0.023*	
C25	0.71282 (10)	0.50604 (10)	0.10674 (6)	0.01502 (19)	
H1N1	0.8472 (16)	0.6975 (16)	−0.0914 (11)	0.028 (4)*	
H2N1	0.9684 (18)	0.6650 (15)	−0.0455 (11)	0.032 (5)*	

### Atomic displacement parameters (Å^2^)

**Table:**

	*U*^11^	*U*^22^	*U*^33^	*U*^12^	*U*^13^	*U*^23^
Sn1	0.01332 (3)	0.00974 (4)	0.01053 (3)	−0.00065 (2)	0.00225 (2)	−0.00024 (2)
O1	0.0199 (3)	0.0093 (4)	0.0152 (3)	0.0003 (3)	0.0032 (3)	0.0005 (3)
O2	0.0169 (3)	0.0107 (4)	0.0122 (3)	−0.0003 (3)	0.0013 (2)	0.0001 (3)
O3	0.0179 (4)	0.0240 (5)	0.0210 (4)	0.0006 (3)	0.0037 (3)	0.0069 (3)
O4	0.0126 (4)	0.0446 (7)	0.0399 (5)	−0.0017 (4)	0.0019 (4)	0.0139 (5)
N1	0.0300 (5)	0.0202 (5)	0.0158 (4)	0.0032 (4)	0.0060 (4)	0.0064 (4)
N2	0.0147 (4)	0.0192 (5)	0.0207 (4)	0.0001 (3)	0.0032 (3)	0.0007 (4)
C1	0.0163 (4)	0.0126 (5)	0.0127 (4)	0.0008 (3)	0.0035 (3)	0.0008 (3)
C2	0.0264 (5)	0.0143 (5)	0.0187 (5)	−0.0024 (4)	0.0080 (4)	−0.0027 (4)
C3	0.0340 (6)	0.0220 (6)	0.0194 (5)	0.0012 (5)	0.0109 (5)	−0.0050 (4)
C4	0.0279 (6)	0.0298 (7)	0.0198 (5)	0.0035 (5)	0.0125 (4)	0.0028 (5)
C5	0.0205 (5)	0.0256 (6)	0.0225 (5)	−0.0029 (4)	0.0076 (4)	0.0041 (5)
C6	0.0179 (4)	0.0177 (5)	0.0169 (5)	−0.0020 (4)	0.0033 (4)	−0.0007 (4)
C7	0.0180 (4)	0.0110 (5)	0.0121 (4)	−0.0022 (4)	0.0025 (3)	0.0001 (3)
C8	0.0170 (4)	0.0239 (6)	0.0164 (5)	−0.0026 (4)	0.0037 (4)	−0.0024 (4)
C9	0.0190 (5)	0.0422 (8)	0.0203 (5)	−0.0086 (5)	0.0014 (4)	−0.0037 (5)
C10	0.0326 (6)	0.0358 (8)	0.0186 (5)	−0.0178 (6)	0.0030 (5)	−0.0081 (5)
C11	0.0426 (7)	0.0173 (6)	0.0176 (5)	−0.0072 (5)	0.0075 (5)	−0.0059 (4)
C12	0.0282 (5)	0.0120 (5)	0.0155 (5)	0.0010 (4)	0.0041 (4)	−0.0004 (4)
C13	0.0152 (4)	0.0144 (5)	0.0174 (5)	−0.0005 (4)	0.0019 (3)	0.0052 (4)
C14	0.0192 (5)	0.0284 (7)	0.0166 (5)	0.0016 (4)	0.0052 (4)	0.0017 (4)
C15	0.0184 (5)	0.0444 (9)	0.0259 (6)	0.0052 (5)	0.0082 (4)	0.0084 (6)
C16	0.0165 (5)	0.0367 (9)	0.0500 (9)	−0.0036 (5)	0.0012 (5)	0.0195 (7)
C17	0.0202 (6)	0.0196 (7)	0.0683 (11)	−0.0053 (5)	−0.0064 (6)	0.0053 (7)
C18	0.0180 (5)	0.0140 (6)	0.0453 (8)	−0.0004 (4)	−0.0020 (5)	0.0000 (5)
C19	0.0123 (4)	0.0114 (5)	0.0124 (4)	−0.0002 (3)	0.0039 (3)	0.0015 (3)
C20	0.0154 (4)	0.0096 (5)	0.0120 (4)	0.0007 (3)	0.0013 (3)	−0.0010 (3)
C21	0.0175 (4)	0.0120 (5)	0.0126 (4)	0.0011 (4)	0.0023 (3)	0.0004 (3)
C22	0.0231 (5)	0.0122 (5)	0.0125 (4)	0.0012 (4)	0.0025 (4)	0.0000 (4)
C23	0.0221 (5)	0.0173 (5)	0.0170 (5)	0.0038 (4)	−0.0017 (4)	0.0031 (4)
C24	0.0163 (4)	0.0194 (6)	0.0202 (5)	0.0030 (4)	−0.0012 (4)	0.0016 (4)
C25	0.0154 (4)	0.0137 (5)	0.0152 (4)	0.0002 (4)	0.0016 (3)	0.0003 (4)

### Geometric parameters (Å, °)

**Table:**

Sn1—C1	2.1129 (11)	C9—C10	1.390 (2)
Sn1—C7	2.1222 (10)	C9—H9A	0.9500
Sn1—C13	2.1239 (11)	C10—C11	1.381 (2)
Sn1—O1	2.2205 (8)	C10—H10A	0.9500
Sn1—O2^i^	2.3345 (8)	C11—C12	1.3949 (17)
O1—C19	1.2651 (13)	C11—H11A	0.9500
O2—C19	1.2563 (12)	C12—H12A	0.9500
O2—Sn1^ii^	2.3345 (8)	C13—C14	1.3965 (17)
O3—N2	1.2315 (13)	C13—C18	1.3978 (18)
O4—N2	1.2344 (12)	C14—C15	1.3968 (17)
N1—C22	1.3616 (15)	C14—H14A	0.9500
N1—H1N1	0.852 (18)	C15—C16	1.385 (2)
N1—H2N1	0.853 (19)	C15—H15A	0.9500
N2—C25	1.4447 (15)	C16—C17	1.390 (2)
C1—C2	1.3928 (16)	C16—H16A	0.9500
C1—C6	1.3972 (16)	C17—C18	1.3947 (18)
C2—C3	1.3952 (17)	C17—H17A	0.9500
C2—H2A	0.9500	C18—H18A	0.9500
C3—C4	1.390 (2)	C19—C20	1.5135 (14)
C3—H3A	0.9500	C20—C21	1.3862 (15)
C4—C5	1.3876 (19)	C20—C25	1.3984 (14)
C4—H4A	0.9500	C21—C22	1.4116 (15)
C5—C6	1.3937 (16)	C21—H21A	0.9500
C5—H5A	0.9500	C22—C23	1.4068 (16)
C6—H6A	0.9500	C23—C24	1.3742 (17)
C7—C12	1.3933 (16)	C23—H23A	0.9500
C7—C8	1.3988 (15)	C24—C25	1.3982 (15)
C8—C9	1.3960 (16)	C24—H24A	0.9500
C8—H8A	0.9500		
			
C1—Sn1—C7	108.43 (4)	C9—C10—H10A	119.9
C1—Sn1—C13	124.55 (4)	C10—C11—C12	119.92 (12)
C7—Sn1—C13	126.79 (4)	C10—C11—H11A	120.0
C1—Sn1—O1	96.31 (4)	C12—C11—H11A	120.0
C7—Sn1—O1	86.85 (4)	C7—C12—C11	120.73 (11)
C13—Sn1—O1	91.67 (4)	C7—C12—H12A	119.6
C1—Sn1—O2^i^	89.71 (4)	C11—C12—H12A	119.6
C7—Sn1—O2^i^	84.74 (3)	C14—C13—C18	119.01 (11)
C13—Sn1—O2^i^	90.55 (4)	C14—C13—Sn1	121.60 (9)
O1—Sn1—O2^i^	170.88 (3)	C18—C13—Sn1	119.37 (9)
C19—O1—Sn1	139.32 (7)	C13—C14—C15	120.28 (13)
C19—O2—Sn1^ii^	132.42 (7)	C13—C14—H14A	119.9
C22—N1—H1N1	119.4 (12)	C15—C14—H14A	119.9
C22—N1—H2N1	120.9 (13)	C16—C15—C14	120.35 (13)
H1N1—N1—H2N1	116.6 (17)	C16—C15—H15A	119.8
O3—N2—O4	122.76 (11)	C14—C15—H15A	119.8
O3—N2—C25	118.84 (9)	C15—C16—C17	119.76 (12)
O4—N2—C25	118.39 (10)	C15—C16—H16A	120.1
C2—C1—C6	119.00 (10)	C17—C16—H16A	120.1
C2—C1—Sn1	122.94 (8)	C16—C17—C18	120.20 (15)
C6—C1—Sn1	117.97 (8)	C16—C17—H17A	119.9
C1—C2—C3	120.43 (11)	C18—C17—H17A	119.9
C1—C2—H2A	119.8	C17—C18—C13	120.40 (14)
C3—C2—H2A	119.8	C17—C18—H18A	119.8
C4—C3—C2	119.97 (12)	C13—C18—H18A	119.8
C4—C3—H3A	120.0	O2—C19—O1	125.29 (10)
C2—C3—H3A	120.0	O2—C19—C20	120.10 (10)
C5—C4—C3	120.16 (11)	O1—C19—C20	114.46 (9)
C5—C4—H4A	119.9	C21—C20—C25	118.85 (9)
C3—C4—H4A	119.9	C21—C20—C19	118.26 (9)
C4—C5—C6	119.71 (12)	C25—C20—C19	122.80 (9)
C4—C5—H5A	120.1	C20—C21—C22	121.02 (10)
C6—C5—H5A	120.1	C20—C21—H21A	119.5
C5—C6—C1	120.71 (11)	C22—C21—H21A	119.5
C5—C6—H6A	119.6	N1—C22—C23	120.48 (11)
C1—C6—H6A	119.6	N1—C22—C21	120.82 (11)
C12—C7—C8	118.86 (10)	C23—C22—C21	118.70 (10)
C12—C7—Sn1	123.56 (8)	C24—C23—C22	120.46 (10)
C8—C7—Sn1	117.46 (8)	C24—C23—H23A	119.8
C9—C8—C7	120.34 (12)	C22—C23—H23A	119.8
C9—C8—H8A	119.8	C23—C24—C25	120.09 (10)
C7—C8—H8A	119.8	C23—C24—H24A	120.0
C10—C9—C8	119.88 (12)	C25—C24—H24A	120.0
C10—C9—H9A	120.1	C24—C25—C20	120.74 (10)
C8—C9—H9A	120.1	C24—C25—N2	118.73 (10)
C11—C10—C9	120.25 (11)	C20—C25—N2	120.48 (9)
C11—C10—H10A	119.9		
			
C1—Sn1—O1—C19	44.23 (11)	O2^i^—Sn1—C13—C14	45.61 (9)
C7—Sn1—O1—C19	152.44 (11)	C1—Sn1—C13—C18	−42.79 (11)
C13—Sn1—O1—C19	−80.80 (11)	C7—Sn1—C13—C18	143.36 (9)
C7—Sn1—C1—C2	−150.32 (9)	O1—Sn1—C13—C18	56.05 (10)
C13—Sn1—C1—C2	34.87 (11)	O2^i^—Sn1—C13—C18	−132.81 (10)
O1—Sn1—C1—C2	−61.55 (10)	C18—C13—C14—C15	−0.60 (18)
O2^i^—Sn1—C1—C2	125.33 (9)	Sn1—C13—C14—C15	−179.01 (9)
C7—Sn1—C1—C6	33.28 (10)	C13—C14—C15—C16	0.5 (2)
C13—Sn1—C1—C6	−141.54 (8)	C14—C15—C16—C17	0.3 (2)
O1—Sn1—C1—C6	122.05 (8)	C15—C16—C17—C18	−0.9 (2)
O2^i^—Sn1—C1—C6	−51.08 (9)	C16—C17—C18—C13	0.8 (2)
C6—C1—C2—C3	1.14 (17)	C14—C13—C18—C17	0.0 (2)
Sn1—C1—C2—C3	−175.23 (9)	Sn1—C13—C18—C17	178.42 (12)
C1—C2—C3—C4	−0.5 (2)	Sn1^ii^—O2—C19—O1	161.05 (8)
C2—C3—C4—C5	−0.5 (2)	Sn1^ii^—O2—C19—C20	−14.22 (14)
C3—C4—C5—C6	0.8 (2)	Sn1—O1—C19—O2	23.95 (17)
C4—C5—C6—C1	−0.13 (18)	Sn1—O1—C19—C20	−160.54 (8)
C2—C1—C6—C5	−0.84 (17)	O2—C19—C20—C21	84.57 (13)
Sn1—C1—C6—C5	175.71 (9)	O1—C19—C20—C21	−91.18 (12)
C1—Sn1—C7—C12	−137.16 (9)	O2—C19—C20—C25	−99.06 (12)
C13—Sn1—C7—C12	37.51 (11)	O1—C19—C20—C25	85.18 (13)
O1—Sn1—C7—C12	127.23 (10)	C25—C20—C21—C22	0.66 (16)
O2^i^—Sn1—C7—C12	−49.23 (9)	C19—C20—C21—C22	177.17 (10)
C1—Sn1—C7—C8	38.72 (10)	C20—C21—C22—N1	−177.84 (11)
C13—Sn1—C7—C8	−146.61 (8)	C20—C21—C22—C23	2.76 (17)
O1—Sn1—C7—C8	−56.89 (9)	N1—C22—C23—C24	177.06 (12)
O2^i^—Sn1—C7—C8	126.65 (9)	C21—C22—C23—C24	−3.54 (18)
C12—C7—C8—C9	0.47 (18)	C22—C23—C24—C25	0.88 (19)
Sn1—C7—C8—C9	−175.60 (10)	C23—C24—C25—C20	2.66 (18)
C7—C8—C9—C10	0.5 (2)	C23—C24—C25—N2	−175.00 (11)
C8—C9—C10—C11	−0.8 (2)	C21—C20—C25—C24	−3.40 (16)
C9—C10—C11—C12	0.1 (2)	C19—C20—C25—C24	−179.75 (10)
C8—C7—C12—C11	−1.23 (17)	C21—C20—C25—N2	174.21 (10)
Sn1—C7—C12—C11	174.60 (9)	C19—C20—C25—N2	−2.13 (16)
C10—C11—C12—C7	0.96 (19)	O3—N2—C25—C24	−173.22 (11)
C1—Sn1—C13—C14	135.63 (9)	O4—N2—C25—C24	7.77 (17)
C7—Sn1—C13—C14	−38.23 (11)	O3—N2—C25—C20	9.12 (16)
O1—Sn1—C13—C14	−125.54 (9)	O4—N2—C25—C20	−169.90 (11)

Symmetry codes: (i) −*x*+2, *y*−1/2, −*z*+1/2; (ii) −*x*+2, *y*+1/2, −*z*+1/2.

### Hydrogen-bond geometry (Å, °)

**Table:**

Cg1 and Cg2 are the centroids of the C1–C6 and C7–C12 phenyl rings, respectively.

**Table:**

*D*—H···*A*	*D*—H	H···*A*	*D*···*A*	*D*—H···*A*
N1—H2N1···O1^iii^	0.85 (2)	2.498 (19)	3.0619 (14)	124.3 (15)
C5—H5A···O4^iv^	0.95	2.40	3.3288 (16)	167
C3—H3A···Cg2^v^	0.95	2.58	3.4430 (14)	152
C21—H21A···Cg1^ii^	0.95	2.70	3.4669 (12)	138

Symmetry codes: (iii) −*x*+2, −*y*+1, −*z*; (iv) −*x*+1, *y*−1/2, −*z*+1/2; (v) *x*, −*y*−1/2, *z*−1/2; (ii) −*x*+2, *y*+1/2, −*z*+1/2.
